# Triple-Negative Breast Cancer: Current Understanding and Future Therapeutic Breakthrough Targeting Cancer Stemness

**DOI:** 10.3390/cancers11091334

**Published:** 2019-09-09

**Authors:** Kha-Liang Lee, Yung-Che Kuo, Yuan-Soon Ho, Yen-Hua Huang

**Affiliations:** 1 Department of Biochemistry and Molecular Cell Biology, School of Medicine, College of Medicine, Taipei Medical University, Taipei 11031, Taiwan (K.-L.L.) (Y.-C.K.); 2 Graduate Institute of Medical Sciences, College of Medicine, Taipei Medical University, Taipei 11031, Taiwan; 3 TMU Research Center for Cell Therapy and Regeneration Medicine, Taipei Medical University, Taipei 11031, Taiwan; 4 TMU Research Center of Cancer Translational Medicine, Taipei Medical University, Taipei 11031, Taiwan; 5 School of Medical Laboratory Science and Biotechnology, College of Medical Science and Technology, Taipei Medical University, Taipei 11031, Taiwan; 6 International PhD Program for Cell Therapy and Regeneration Medicine, College of Medicine, Taipei Medical University, Taipei 11031, Taiwan; 7 Center for Reproductive Medicine, Taipei Medical University Hospital, Taipei Medical University, Taipei 11031, Taiwan; 8 Comprehensive Cancer Center of Taipei Medical University, Taipei 11031, Taiwan; 9 Ph.D. Program for Translational Medicine, College of Medical Science and Technology, Taipei Medical University, Taipei 11031, Taiwan

**Keywords:** TNBC, Recurrence, Resistance, Cancer stem cell, IGF-1R, nAChR

## Abstract

Triple-negative breast cancer (TNBC) is cancer that tested as negative for estrogen receptors (ER), progesterone receptors (PR), and excess human epidermal growth factor receptor 2 (HER2) protein which accounts for 15%–20% of all breast cancer cases. TNBC is considered to be a poorer prognosis than other types of breast cancer, mainly because it involves more aggressive phenotypes that are similar to stem cell–like cancer cells (cancer stem cell, CSC). Thus, targeted treatment of TNBC remains a major challenge in clinical practice. This review article surveys the latest evidence concerning the role of genomic alteration in current TNBC treatment responses, current clinical trials and potential targeting sites, CSC and drug resistance, and potential strategies targeting CSCs in TNBC. Furthermore, the role of insulin-like growth factor 1 receptor (IGF-1R) and nicotinic acetylcholine receptors (nAChR) in stemness expression, chemoresistance, and metastasis in TNBC and their relevance to potential treatments are also discussed and highlighted.

## 1. Introduction

Breast cancer is the most frequently diagnosed malignancy and the main cause of cancer-related death in women. Globally, approximately 2.1 million new female breast cancer cases and 627,000 deaths were estimated to have occurred in 2018 [1]. Recurrence and metastasis are the major cause of these deaths. Approximately 15%–20% of these patients could be classified as “triple-negative.” The diagnosis is made by observing exclusion of the expression and/or amplification of three biomarkers (estrogen receptor [ER], progesterone receptor [PR], and human epidermal growth factor receptor 2 [HER2] protein) [2].

So-called triple negative breast cancer (TNBC) is more commonly diagnosed in women younger than 40 years compared to hormone-positive breast cancer [3]. The histology is usually high grade and most commonly interpreted as infiltrating ductal carcinoma, which exhibits geographic necrosis, a pushing border of invasion, and a stromal lymphocytic response. Clinically, TNBC tends to act more violently than other types of breast cancer and is characterized by a high risk of relapse, short progression-free survival (PFS), and overall survival (OS) [4]. One-half of patients with early-stage TNBC (stages I to III) experience disease recurrence, and 37% of patients experience a 5-year mortality rate after surgery [5]. Also, patients with metastatic TNBC have short PFS after failure of first-line chemotherapy (median PFS, 3 to 4 months), suggesting the highly unmet need for the development of a drug targeting TNBC [6].

TNBC is clinically heterogeneous, with deviations in morphology, mutational phenotype, and signaling profiles between tumors. Notably, the diagnostic criteria have not been developed to distinguish a distinct biologic subtype of breast cancer—a “triple-negative” phenotype. A histologic subtype, medullary carcinoma, despite generally being triple-negative, has a very good prognosis [7]. Due to next-generation sequencing, our understanding of the heterogeneity of TNBC is evolving. For example, TNBC can be clustered into at least six subtypes on the basis of gene ontologies and expression profiling: basal-like 1, basal-like 2, immune modulator, mesenchymal, mesenchymal stem–like, and luminal androgen receptor [8]. Additional subtypes include claudin-low and interferon-rich subtypes [9,10]. Furthermore, the tumor suppressor gene p53 (*TP53*) and several DNA repair genes, particularly the *BRCA* genes, are either mutated or abnormally expressed in TNBC. These molecular characteristics may influence chemotherapy sensitivity to direct DNA-damaging agents such as platinum [11].

## 2. Molecular Classification of TNBC

In 2000, Perou et al. discovered a classification of breast cancer based on gene expression patterns. The triple-negative clinical phenotype mostly comprises the basal cell–like subgroup [12]. However, triple-negative and basal cell breast cancers are not synonymous. Immunohistochemical (IHC) and molecular profiling studies have suggested that only a subgroup of TNBC expresses the combination of basal cell markers (for instance, CK5 and CK14) [13]: both categories have up to 30% discordance [14]. In addition, basal-like can further divide into KRT5/6^+^, EGFR^−^, and c-KIT^−^ subgroups [15].

During the last decade, numerous studies have developed exclusive molecular classifications for TNBC. Rody et al. first distinguished a molecular subgroup by defining 16 metagenes within the group [16]. Later, Lehmann et al. identified seven molecular subgroups: unstable cluster (UNS), basal-like 1 (BL1), basal-like 2 (BL2), immunomodulatory (IM), mesenchymal (MES) like, mesenchymal stem like (MSL), and luminal androgen receptor (LAR) [8]. In addition, in another intrinsic subgroup, approximately 70% of claudin-low tumors are TNBC, with a high frequency of metaplastic and medullary differentiation [2,10]. The IM and MSL subtypes have since been refined [17]. Burstein et al. utilized nonnegative matrix factorization and defined four subgroups: basal-like immune active, basal-like immune suppressed, mesenchymal, and luminal AR [18]. Another study showed basal A, basal B, basoluminal, and luminal subtypes existing in TNBC [19]. Most recently, Prado-Vazquez et al. applied probabilistic graphical models to explore the molecular analysis of TNBC from the perspective of a CSC hypothesis. They proposed at least two independent biological levels—cellular and immune—to stratify the prognostic and possible therapeutic classification [20]. The aforementioned subtypes display distinct therapeutic responses and pathological complete response (pCR) rates after neoadjuvant chemotherapy [21]. In the Lehmann classification, cell cycle and DNA damage response genes are highly expressed in BL1 tumors, with a high mitotic index. Clinically, patients with BL1 subtypes exhibit good response to antimitotic agents such as taxanes (paclitaxel or docetaxel) and the DNA-damaging agent cisplatin, achieving approximately one half of pCR rates after neoadjuvant chemotherapy. Additionally, survival-mediated receptor tyrosine kinases, proliferation genes, and metabolic signaling genes are enriched in BL2 tumors. These patients, however, seldom achieve a pCR. MSL subtypes are sensitive to sarcoma family kinase (SRC) and phosphoinositide 3-kinase (PI3K)/mammalian target of rapamycin (mTOR) inhibitor tumors and thus have moderate pCR rates (23%–31%). In addition, expression of epithelial–mesenchymal transition (EMT) markers is enhanced in the MES and MSL subtypes, with low expression levels for proliferation-related genes and accompanied by a low mitotic index [8]. Furthermore, transforming growth factor *β* (TGF-*β*) and receptor type III (TGFBRIII) were demonstrated to drive migration and invasion of MSL cell lines both in vitro and in vivo [22]. These studies clearly demonstrated that in patients receiving the same treatment, the heterogeneous nature of the effect of molecular and genomic expression was evident. Recently, a Phase Ib/II, open-label, umbrella study (NCT03805399) was conducted to evaluate multiple targeted treatments in patients with specific subtypes of TNBC according to Fudan University Shanghai Cancer Center 500+ gene panel testing and IHC subtype staining. Despite these extensive studies, the clinical effect of the designation of TNBC molecular subtypes remains largely undetermined, and future studies are required.

## 3. Response to Treatment: Clinical Practice and Genomic Alteration of TNBC

Treatment for TNBC remains a major clinical challenge due to the lack of causally proven oncogenic drivers through which to target the vast disease heterogeneity [8,23]. To date, the principles for the surgical management of and radiation therapy options for TNBC are applied in a similar manner across various breast cancer subtypes. In nonmetastatic settings, neoadjuvant or adjuvant chemotherapy is typically administered for women with TNBC ≥0.5 cm or node-positive TNBC (regardless of tumor size). Typically, tumor size, lymph node status, grade, overall performance status, and the presence or absence of medical comorbidities determines the chemo regimens used. These patients have a higher risk of relapse compared with other breast cancer phenotypes and are not candidates for other forms of adjuvant therapy (i.e., HER2-directed treatment or endocrine therapy) [5,24]. Interestingly, in patients with metastatic breast cancer, there is a possible discordance of ER, PR, and HER2 markers between primary and metastatic disease—12.6%, 31.2%, and 5.5%, respectively (*p* < 0.001). Thus, a confirmatory biopsy of a suspected lesion should be obtained when possible [25].

Because patients with TNBC commonly do not achieve a pCR following chemotherapy, the selection of chemotherapy to use against different TNBC subtypes is being debated [21]. Neoadjuvant anthracycline-based chemotherapy is related to a higher pCR in TNBC compared with luminal non-TNBC subtypes and is therefore reasonable to consider. In the adjuvant therapy space, the principles for non-TNBC apply equally to TNBC, and these can include administering anthracyclines, taxanes, and/or platinum compounds to disrupt cancer cell survival [5,26,27]. The addition of platinum compounds to standard chemotherapy has doubled pCR rates in patients with TNBC [26], but those who fail to achieve pCR exhibit worse outcomes compared with other subtypes of breast cancer [5].

Several studies, including in vitro and in vivo studies and clinical trials, have defined genomic effects inherent to TNBC response to treatment. Silver et al. demonstrated that the alteration of *BRCA1* expression, caused by promoter methylation and *p53* mutations, conferred good prognosis to cisplatin treatment [27]. Similarly, *CD73* expression has been associated with doxorubicin resistance in patients with TNBC [28]. Another study focusing on genomic adaptations in basal-like tumors revealed mutations of *p53* and *PIK3CA*, loss of *PTEN* and *RB1*, amplification of *cyclin E1,* and increased expression of *MYC* and *HIF1-α* [29]. Furthermore, Balko et al. analyzed residual breast cancer after neoadjuvant chemotherapy and identified the additional amplification of several genes (*CDK4*, *MCL1*, *JAK2*, *AKT1,* and *EGFR*) and loss of mutations in *BRCA1/2*, *ATM*, and *CDKN2A* [30]. These findings have encouraged more research efforts to identify effective therapeutic strategies for TNBC.

## 4. Current Clinical Trials in Triple-Negative Breast Cancer

**Figure 1:**
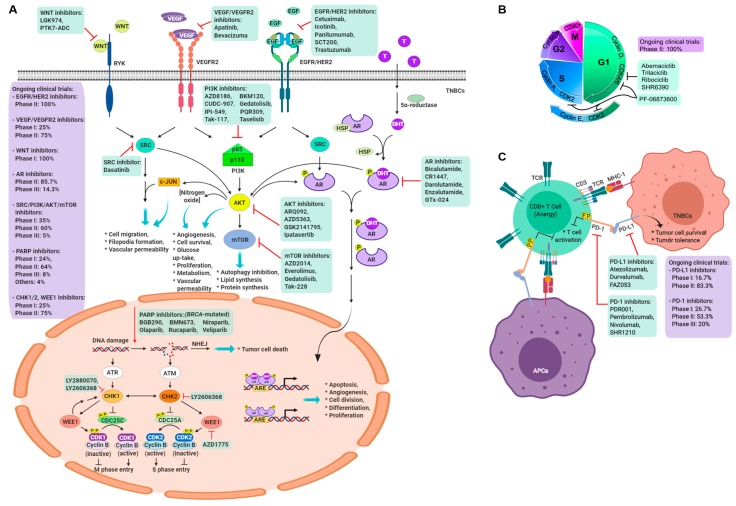
Diagram of ongoing clinical trials in TNBCs. (**A**) System view of signaling pathways activated by extracellular stimuli, which triggered multiple biological functions through central hub AKT or subsequent effectors. Numerous inhibitors attempt to attack these critical molecules in TNBCs, and ongoing phases of clinical trials for categorized inhibitors have also revealed the same. (**B**) Disruption of cell cycle by CDK inhibitors in TNBC trials. (**C**) Blockade of immune checkpoints in cancer immunotherapy. Prevention of PD-1/PD-L1 signaling transduction from inhibitors could activate CD8^+^ T cells to kill TNBC tumor cells.

A summary of current trials of single-agent treatments or combinations of different target therapeutic reagents and chemotherapy is provided in Table 1. We describe these targets and treatments in a cell function–based manner, emphasizing DNA repair and damage, growth factor and angiogenesis, specific hormone receptors, and immune molecular checkpoints (Figure 1).

## 5. Targeting the BRCA1/2 Pathway

### 5.1. PARP Inhibitors

Inhibition of *PARP1* could induce double-strand DNA breaks, where DNA damage is repaired through a BRCA pathway–dependent homologous recombination mechanism under normal circumstances. Therefore, inhibition of PARP is particularly useful in BRCA-mutated breast cancer with aberrant DNA damage responses (DDR) [31,32,33]. Given the shared clinicopathologic characteristics of *BRCA*-mutated breast cancer and TNBC, the efficacy and safety of PARP inhibition is being tested in both settings. Among the subset of 121 *BRCA* mutation carriers with metastatic triple-negative disease in the OlympiAD trial has been grouped to anthracycline and a taxane in either the adjuvant or metastatic setting. Those that were randomly assigned to olaparib exhibited improved PFS relative to those receiving chemotherapy (HR for progression or death 0.43, 95% CI = 0.29–0.63) [34,35]. Notably, the study also indicated positive results for patients with hormone receptor–positive, HER2-negative disease; however, the improvements associated with olaparib were stronger in the triple-negative population. To date, several other PARP inhibitors are in clinical development [34,35,36,37,38,39,40,41,42].

### 5.2. Growth Factors and Angiogenesis

The epidermal growth factor receptor (EGFR/HER1) may be the most well-known protein that is overexpressed in cancers, and for which several monoclonal antibodies and small-molecule inhibitors exist. Several phase II clinical trials have assessed the efficacy of cetuximab, an anti-EGFR monoclonal antibody, which has exhibited modest activity in combination with chemotherapy against advanced TNBC [43,44]. In addition, VEGF and VEGFR that play roles in angiogenesis are considered an important target for cancer therapy. However, to date, prospective studies have not shown that the incorporation of angiogenesis inhibitors has an effect on OS for women with TNBC. Of agents in this class, VEGF antibody (bevacizumab) has been the most widely studied. Unfortunately, research results have consistently suggested that although incorporation of bevacizumab can improve PFS, it has virtually no effect on OS [45,46,47,48,49].

### 5.3. Androgen Receptor Inhibitor

Both normal and malignant breast tissue express the androgen receptor (AR) [50]. Luminal hormone receptor–positive breast cancers more commonly express AR (91%), TNBC expresses AR approximately 30% of the time [51]. Interestingly, prognosis for those with AR-positive TNBC has been shown to be more favorable than for those with non-AR-positive TNBC [8]. Several studies have sought to define the antitumor activity of AR inhibition in advanced TNBC. Gulcap et al. reported a six-month clinical benefit rate (CBR) of 19% (95% CI = 0.07–0.39) for the AR antagonist bicalutamide among 50 patients (12% of 424 screened) with metastatic AR-positive TNBC [52]. The potent AR inhibitor enzalutamide was evaluated in 118 patients with AR-positive metastatic TNBC (55% of 404 screened) [53]. Overall CBR at 16 weeks was 25% (95% CI = 0.17–0.33) and was 33% (95% CI = 0.23–0.45) among those whose tumors expressed ≥10% nuclear AR. Although this remains a treatment strategy of unknown importance, it is clear that optimizing a robust biomarker and/or signature for those most likely to respond to AR inhibition will be the key to pursuing it. Moreover, the inherently favorable biology of AR-positive advanced TNBC may also play a role in the apparent benefit from AR inhibition.

### 5.4. Immune Checkpoint Molecule Inhibitor

Immunologic escape describes the stage at which malignant clones acquire the capability of evading the adaptive immune system. Programmed cell death 1 (PD-1) is a transmembrane protein expressed on T cells, B cells, and NK cells: PD-1 ligand (PD-L1) binds to PD-1 and then directly inhibits apoptosis of the tumor cell while promoting peripheral T effector cell exhaustion and conversion of T effector cells to Treg cells [54]. In addition, PD-L1 is expressed on the surface of multiple tissue types, including many tumor cells, hematopoietic cells, and PD-1 ligand 2 (PD-L2) restricted hematopoietic cells. Tumors can upregulate the expression of PD-1 and PD-L1, which promotes peripheral T cell exhaustion [55]. Evidence that this approach may be useful in early breast cancer comes from a small neoadjuvant trial that discovered improved pCR when the anti-PD-1 antibody pembrolizumab was added to anthracycline/taxane-based chemotherapy [56]. Early clinical experience with immunotherapy for TNBC also includes small studies of the single-agent anti-PD-1 antibody pembrolizumab as well as anti-PD-L1 antibodies avelumab and atezolizumab, with response rates generally lower than 20%, even in PD-L1-selected tumors [57,58]. In a phase III randomized trial (IMpassion 130 trial), 902 patients who had not received treatment for metastatic TNBC were randomly assigned to receive nab-paclitaxel with either atezolizumab or a placebo [59]. Overall, there was only a modest but statistically significant difference in PFS (7.2 vs. 5.5 months; HR 0.80, 95% CI = 0.69–0.92) in favor of incorporating atezolizumab. However, in a prospectively planned subset analysis of outcomes according to PD-L1-expressing immune effector cells within the tumors, atezolizumab improved both PFS (7.5 vs. 5.0 months; HR 0.62, 95% CI = 0.49–0.78) and, importantly, OS (25.0 vs. 15.5 months; HR 0.62, 95% CI = 0.45–0.86). Additional strategies, including combining immunotherapy with other systemic therapy or radiation, as well as other approaches, are currently in development (Figure 1C and Table 1). Furthermore, the optimization of biomarkers predictive of response to immunotherapy is actively under investigation.

## 6. CSCs and Drug Resistance in TNBC

### 6.1. Expression of Cancer Stemness

In recent decades, the theory of CSCs or cancer stemness has captured much attention and interest. It is thought that this theory has potential to revolutionize our understanding of the cellular and molecular events during cancer progression and how they contribute to drug resistance, tumor recurrence, and metastasis [60]. Among solid tumors, breast CSCs (BCSCs) were the first to be identified. An extremely small number of BCSCs were able to produce tumors in immune-deficient NOD/SCID mice [61]. Conventional cancer therapies against the majority of cancer cells are insufficient to eradicate all cells within the tumor, especially CSCs, due to their highly resistant nature, leading to tumor recurrence and distant metastasis [62]. Two types of CSCs with resistant ability need to be addressed: quiescent (non–proliferative) CSCs and proliferative CSCs. These cell types express stemness properties, resistant features, metastasis and immune evasion, but could be discriminated from rate of cell cycle [63]. However, the consequence of quiescent CSCs for anti-mitotic chemodrugs treatment is different from proliferative CSCs. The proliferative CSCs would be killed by anti-mitotic agent with higher dosage than general tumor cells, but quiescent CSCs are still surviving in the highly dosage level. Indeed, after initially chemotherapeutic treatment, resident quiescent CSCs cause tumor relapse when they evoke by suitable niche signals [64]. Given dynamic cellular events during cancer progression and CSC-mediated resistance, understanding the heterogeneous nature of their regulatory mechanism provides a concrete foundation for the development of CSC-specific therapeutics.

In addition, another cell type, quiescent non-CSCs, also resists anti–mitotic drugs. In primary tumor, growth phases transition between proliferation and dormancy in non-CSCs are determined by genetic and epigenetic alternations [63,65]. Drug resistance remains a continuing challenge in cancer treatment and is undoubtedly the major reason for treatment failure [66,67]. Multidrug resistance (MDR) is a phenomenon that describes common clinical drug resistance to a broad spectrum of drugs [68,69]. For the proliferative CSCs, two types of drug resistance have been discussed: (1) acquired resistance in response to treatment and (2) intrinsic resistance, where resistance is to a spectrum of drugs, even when the drug is freshly used against a specific tumor [67,70].

The concept of cancer stemness or CSCs is currently used to explain the mechanism of MDR. These cells are recognized as a group of cells that possess much higher endogenous resistance against radiation and chemotherapy than non-CSC differentiated tumor cells [71]. CSCs and normal stem cells (SCs) seem to share similar drug resistance abilities [72]. Because the pool of cells in an organism are maintained by SCs, it is necessary to maintain and protect these SCs biologically. Thus, several mechanisms have been developed to maintain cell stemness and avoid death by apoptosis. CSCs appear to apply these intrinsic “gadgets” against anticancer therapies. As a result, chemotherapy and radiotherapy treatment eradicate the majority of the population of non-CSCs but not CSCs, consequently leading to recurrence of the disease [73].

The study of CD44/CD24 and ALDH1 expression could be the most appropriate to identify BCSCs with distinct levels of differentiation from breast cancer populations. In an analysis of 466 invasive breast carcinomas and eight breast cancer cell lines, basal-like breast cancer harbored the highest percentage of tumor cells with the CSC phenotype CD44^+^CD24^−/low^ and ALDH1 positivity [74]. Clinically, their expression is associated with worse chemotherapy response, lymph node metastasis, distant metastasis, recurrence, and worse disease-free survival (DFS) and OS [75,76]. Enriched ALDH1-expressing cells are an independent prognostic factor that predicts poor prognosis in patients with TNBC [77,78,79]. Furthermore, the expression of STAT3 acts as a promising chemoresistance biomarker and is also associated with the CD44^+^/CD24^−/low^/ALDH^+^ BCSC-like subset of the TNBC cell line [80]. In patients with TNBC, front-line chemotherapy effectively suppresses the bulk of primary tumors by eradicating proliferating cells but commonly fails to target the slow-cycling CSCs. Thus, identifying molecular drivers and signaling pathways that govern the self-renewal and expansion of CSCs has the potential to inspire new treatment options for this lethal disease. We herein discuss several mechanisms of MDR in TNBC against concurrent chemo regimens such as anthracyclines, taxanes, and/or platinum [5,26,27].

### 6.2. Cancer Niche and Therapy Resistance

CSCs are located in a specialized microenvironment termed the niche that is mainly composed of cancer-associated fibroblasts (CAF), endothelial cells (ECs), mesenchymal SCs (MSC), tumor-associated macrophages (TAM), and extracellular matrix (ECM), which play different roles in orchestrating therapy resistance [81,82,83]. Secretion of several cytokines and chemokines, such as IL6/IL8, CXCL12, and CXCL7, in CSC niches activate the CSC signaling network and the NF-κB pathway, leading to CSCs characteristics and furthering therapy resistance [72]. Studies have demonstrated that CAF coculture increases the secretion of type I collagen, contributing to decreased drug uptake in breast cancer cell lines [84,85]. In addition, interaction between CAF and immune cells induces self-renewal properties through activation of NF-κB signaling in breast, gastric, prostate, and glioma CSCs [86]. CAF are able to secret exosomes through the receptor RIG-1 and activate STAT1 in breast cancer cells; in turn, STAT1 activation further activates NOTCH3, leading to increased drug resistance in CSCs [87]. MSCs are adult SCs that under normal conditions act as immunomodulators. However, similar to CAF, physical interaction between MSCs and breast cancer enhances resistance to trastuzumab through activation of the non-receptor tyrosine kinase Src and its downstream PI3K/Akt pathway [83]. Additionally, the interaction with MSCs mediates the acquisition of MDR proteins and also increases the resistance of epithelial ovarian cancers to carboplatin and paclitaxel [88]. The vascular microenvironment has been related to the maintenance of a self-renewing CSC pool in brain tumors since 2007 [89]. ECs secrete TNFα, which activates the NF-κB signaling pathway in CSCs and induces the secretion of several factors, including CXCL1/2 in breast cancer; the process works through the attraction of immune cells and production of chemokines, including S100A8/9, which consequently induces chemoresistance to doxorubicin and cyclophosphamide. Furthermore, the resistance can be suppressed by CXCR2 blockers [90]. Immune cells such as TAMs interact with cancer cells through a variety of growth factors, cytokines, and chemokines [91,92]. For example, IL6 and STAT3 pathways are related to trastuzumab resistance in BCSCs [93]. The ECM is an assembly of molecules mostly secreted by fibroblasts. Increased ECM stiffness in solid tumors protects CSCs from chemotherapeutic agents resulting from physical barriers that separate the cells from any chemotherapeutic effect [94]. Moreover, the ECM interacts with CSCs, and regulates stem and proliferative signaling pathways as well as drug resistance. For example, hyaluronic acid is the ligand of the CD44 receptor, which mediates the acquisition and maintenance of CSCs upon interaction [95]. Tenascin C, which is expressed in ECM, improves the efficacy of the Wnt and Notch signaling pathways, thereby stabilizing BCSCs [96].

### 6.3. Cell Membrane Transporters: ABC Family

The ATP-binding cassette (ABC) transporters belong to a family of 49 membrane proteins usually related to the efflux of small molecules and compounds from the cytosol to the extracellular medium using ATP hydrolysis. Due to their ability to expel toxic chemicals, they participate in the development of MDR [97,98]. Many of the human ABC proteins are efflux transporters, and three of them, namely P-glycoprotein (P-gp/MDR1, gene symbol *ABCB1*), the multidrug resistance protein 1 (MRP1, gene symbol *ABCC1*), and the breast cancer resistance protein (BCRP, gene symbol *ABCG2*), have been implicated as major efflux transporters responsible for multidrug resistance in cancer cells [99]. Many ABC transporters are expressed on the surface of CSCs [100]. Thus, staining a population of cells with Hoechst 33342 dye and Rhodamine 123 dye could identify CSC subpopulations within a tumor: This method of identification is due to these dyes being pumped out of CSCs, which can then be identified as the unstained subpopulation—so-called side population (SP) cells—in flow cytometry [101,102,103]. Notably, promoters of ABC transporters carry several binding sites for EMT-inducing transcription factors [104]. In breast cancer, one study reported a correlation between *ABCG2* mRNA expression and response in a subgroup of patients receiving anthracycline-based chemotherapy (5-fluorouracil, adriamycin/epirubicin, and cyclophosphamide), and such a correlation did not exist in the cyclophosphamide, methotrexate, and 5-fluorouracil-treated group of patients [105]. A search of the “GEO Profiles database” revealed a functional genomic aberration of *ABCB1* genes in five paclitaxel-resistant TNBC cell lines (data accessible at NCBI GEO database, accession No. GSE90564). Furthermore, a link between ABCB1 and the Hedgehog pathway supports the relationship between CSCs and ABC transporters [106].

### 6.4. Epithelial-mesenchymal Transition (EMT)

The mechanisms responsible for EMT and CSC-related drug resistance remain uncertain in most cases. One hypothesis suggests that cells undergoing EMT enter into a quiescent state and no longer divide [107]; however, most conventional treatments target actively dividing cells [72]. For example, in oral cancer cells, a SNAIL-mediated EMT phenotype exhibited quiescence and further induced resistance to chemotherapeutics [108]. Similarly, overexpression of TWIST, SNAIL, and the FOXC2-mediated EMT phenotype in TNBC cells led to MDR by upregulating ABC transporters [104]. In summary, EMT activation also promotes stemness and quiescence, which induce drug resistance in multiple cancers.

### 6.5. Hypoxia and ROS

The hypoxic niche plays an important role in the maintenance of SC characteristics during embryonic development and self-renewal [109,110]. In fact, CSCs are typically situated near hypoxic areas within tumors [111]. Hypoxia-inducible factor-1*α* (HIF-1*α*) is the main regulator of cellular responses to hypoxia. It is ubiquitinated at high oxygen levels and subsequently degraded. At decreased oxygen levels, HIF-1*α* is activated upon ubiquitination inhibition, translocates into the nucleus, dimerizes with HIF-1*β*, and activates the transcription of specific genes [112]. HIF-1*α* has long been recognized as a pivotal orchestrator of cancer cell response to hypoxic microenvironments by regulating the expression of over 60 genes involved in metabolic reprogramming and pH balance, cell proliferation/survival, apoptosis, angiogenesis, SC maintenance, matrix remodeling, metastasis, and resistance to radiotherapy and chemotherapy [113,114]. Exposure of TNBC cells to hypoxia has been shown to increase the percentage of BCSCs in a HIF-1*α*–dependent manner [115,116], which also contributes to multiple steps in the metastasis of TNBCs [117]. Clinically, in patients with breast cancer, HIF-1*α* overexpression identified by immunohistochemistry of tumor biopsies has been associated with increased mortality [118]. Increased expression of hypoxia-induced genes in breast cancer is also associated with poor prognosis [119]. Furthermore, HIF-1*α* expression has been reported to be correlated with MDR in breast cancers. For example, expression can be induced by treatment of breast cancer cells with doxorubicin under normoxic environments [120]. Further study revealed that in paclitaxel- or gemcitabine-treated TNBCs, the induction of HIF activity is required for enrichment of BCSCs both in vitro and in vivo [121]. Through the activation of HIF-1*α*, the expression of EMT and stemness activators such as WNT, Hedgehog, and NOTCH pathways, as well as other stemness markers such as FOXA2, cMET, CD133, NANOG, SOX2, SOX17, and PDX1 occurs [122,123]. Because hypoxia results in an unfavorable condition for cellular growth, it induces quiescence in cancer cells [124,125]. Under normal conditions, reactive oxygen species (ROS) accumulation leads to apoptosis in both normal and cancer cells [126,127], whereas the HIF-1*α* signaling pathway decreases the production of ROS and preserves SC properties leading to drug resistance in CSCs [72,128]. In mammospheres composed almost entirely of stem cells, suppressed levels of ROS were observed; this explains the higher rate of radioresistance when compared with differentiated adherent cells [129,130].

Aldehyde dehydrogenase (ALDH) is another molecule responsible for ROS decrease and is also a CSC marker [131]. It comprises a family of 19 cytosolic enzymes involved in intracellular aldehyde oxidation and in the oxidation of retinol to retinoic acid during the early stages of SC differentiation [132,133]. ALDH1, the main isoform, directly reduces ROS and produces antioxidant compounds such as NADP that further facilitates detoxification. In hematopoietic malignancies, ALDH expression is high in quiescent cells [134,135,136]. In addition, ALDH-positive tumors in cancers of the colon, breast, lung, pancreas, bladder, prostate, and ovary are tumorigenic and resistant to chemotherapy [134]. Further, ALDH1 also protects cells against alkylating agents such as paclitaxel [137]. Clinically, more than half of TNBC cases exhibit ALDH1 expression. The enriched ALDH1-expressing cells are an independent prognostic factor that predicts poor prognosis in patients with TNBC [77,78,79]. For the 234 patients treated with neoadjuvant chemotherapy, the pCR rate was significantly lower in ALDH1(+) cases (13.5% vs. 30.3%, *p* = 0.003). The pCR rate and ALDH1 expression were significantly correlated in patients with TNBC (*p* = 0.003). ALDH1(+) breast cancers tend to be aggressive with poor prognoses [138].

### 6.6. High Survival Capacity of CSCs

CSCs are multipotent SCs responsible for the long-term clonal maintenance and growth of most human neoplasms. They can coexist in a cycling and quiescent state as they acquire mutations that further increase heterogeneity [139]. CSCs of the lung, pancreas, glioma, and breast possess highly active DDR systems [71,130]. In CD44^+^CD24^–/low^ BCSCs, DNA single-strand break repair is particularly active and linked to APE1 upregulation [140]. In addition to DNA repair systems, studies have suggested that the mutation or inactivation of cell cycle–regulating genes and apoptosis-inducing genes help CSCs to escape apoptosis [141]. The most well-known example, the loss of p53 function in colon, breast, and lung carcinoma, promotes SNAIL-mediated EMT expression, resulting in increased radioresistance [141]. Compared with their relatively more differentiated counterparts, BCSCs exhibit reduced levels of oxidative DNA damage, both at baseline and after irradiation [130]. EZH2 participates in histone methylation and is also involved in the expansion of CD44^+^CD24^–/low^ BCSCs by epigenetically repressing DNA repair [142]. The basal activation status of checkpoint kinases (rather than the hyperactivation of DNA repair) may also constitute a key mechanism by which CSCs resist genotoxic agents. In studies of human breast tumors, the EMT-inducing transcription factor ZEB1promotes expression of CHK1, which is required to mediate the ATM-dependent G_2_-M checkpoint to survive radiotherapy and eventual metastatic relapse. This result was further established following the inhibition of CHK1, which resensitized the cells [143]. Furthermore, the abrogation of AKT1 signaling increases the radiosensitivity of BCSCs by inhibiting WNT signaling [144]. Collectively, multiple processes have been targeted to force differentiation or reduce DDR signaling and thus increase CSC sensitivity to DNA damage. These results indicate that DDR also stands out as a promising therapeutic target for BCSC eradication.

## 7. Potential Targeting Strategies against CSCs in TNBC 

Several clinical trials have been designed that combine conventional chemotherapy and adjuvant therapies in the hope of eliminating both actively dividing cells and CSCs. These CSC-targeted adjuvant regimens may either attack stem-related pathways or enhance the drug sensitivity of CSCs in distinguished ways (Table 2). In the following, we present intriguing preclinical results that describe many therapeutic strategies that specifically target BCSCs.

## 8. Targeting CSCs

### 8.1. Targeting CSC Specific Marker

Previous studies have revealed that increased cyclophosphamide sensitivity of lung cancer cells could be achieved by knockdown of two ALDH isoforms [145]. As previously mentioned, high levels of ALDH are expressed in CSCs, decreasing levels of ROS and protecting the cells from ROS-mediated DNA damage and subsequent apoptosis [146]; this indicates the potential of ALDH-targeting treatments. Another example is the targeted delivery of iron oxide nanoparticles to CD44^+^ cells for the selective killing of BCSCs using conventional chemotherapy [147].

### 8.2. Targeting CSC Signaling

Due to limited specific markers, several studies have tried to eliminate CSCs by targeting highly activated signaling pathways in these cells. A number of signaling pathways have been associated with the therapy-resistant phenotype of BCSCs, including Notch, Hedgehog, and Wnt, which regulate apoptosis escape, maintenance of a stem cell niche, and increased invasion capacity [148,149,150]. One example of a relatively favorable result concerns basal cell carcinoma; here, the Hedgehog pathway inhibitor, vismodegib, was used. In phase II clinical trials, vismodegib treatment improved one-year median survival in comparison with patients receiving a standard treatment [151,152]. Dasatinib is a potent, oral SRC-family kinase inhibitor with preclinical antiproliferative, antimetastatic, and antiosteoclastic activity in triple-negative or basal-like breast cancer cell lines. However, single-agent dasatinib has limited activity in unselected patients with TNBC. Thus, future studies should investigate repositioning dasatinib in other breast cancer settings, including chemotherapy combinations [153]. Recent in vitro studies have reported an induction of the Wnt pathway in a compensatory fashion when a TNBC cell line is challenged with pan-PI3K inhibition (buparlisib). Essentially, dual PI3K and Wnt pathway inhibitors work as a synergistic combination against cell viability and enhance antitumor efficacy in TNBC cell lines [154]. This approach suggests a paradigm in which targeting both pathways (genomically-aberrant and compensatory) can induce synergistic effects from inhibitors.

### 8.3. Targeting CSC Dormancy and Proliferation

**Figure 2:**
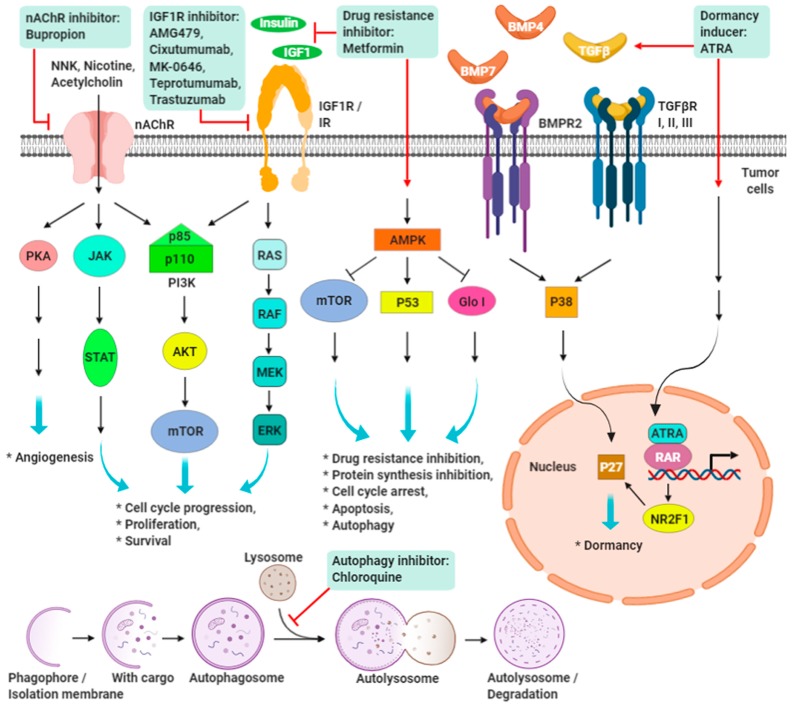
Diagram of ongoing clinical trials of potential targeting strategies against CSCs in carcinoma. System view of signaling pathways activated by extracellular stimuli, which triggered multiple biological functions through subsequent effectors. Numerous inhibitors and inducer attempt to attack either these critical molecules or pathways in CSCs in several types of carcinoma.

Cancer cell dormancy is attributable to G_0_ cell cycle arrest at the single-cell level, which resembles CSCs in primary tumors [155]. The process can be achieved by activating quiescence signaling as a response to new signals in the niche or from the loss of dependent background signals—for instance, hypoxia [146]. To date, the mechanisms underlying local cancer cell dormancy remain largely unknown. Several pharmacological strategies have, therefore, been suggested (i) to maintain cancer cells in the dormant state, (ii) to reactivate dormant cells to increase their sensitivity to anti-proliferative drugs, and (iii) to eliminate dormant cancer cells [155]. The concept of maintaining cancer dormancy first emerged from hormone-deprivation therapy in hormone-dependent cancers; for example, ER+ breast cancer. Today, standard adjuvant anti-estrogen therapy with tamoxifen or the aromatase inhibitor letrozole have been shown to suppress outgrowth of dormant cancer cells and to improve the survival of patients with breast cancer [156,157]. Similarly, dormancy of disseminated breast cancer was able to be maintained with the ERK inhibitor U0126 or the Src inhibitor PP1 [158]. Inhibitors of CDK4/6, which mediate the transition from the G_0_/G_1_ phase to the S-phase of the cell cycle, induce reversible or irreversible G_0_/G_1_ cell cycle arrest in various cancer models [159,160]. Currently, several clinical trials of CDK4/6 inhibitors have been conducted in TNBC (Figure 1B and Table 1). In addition, administering the factors of the premetastatic dormant niche, such as GAS6, BMP4, and BMP7, as well as TGF-*β*2 [161,162,163], has been shown to maintain dormancy in disseminated tumor cells (Figure 2). Most recently, Puig et al. demonstrated that upregulation of the epigenetic modification enzyme TET2 and its catalytic product 5hmC are biomarkers of slow–cycling cancer cell, a cell in dormancy state with chemoresistance, in different cancer types. Targeting TET2 would reverse dormant cell to proliferative cell and diminish chemoresistance [65]. An awakening strategy that forces the cell cycle to operate has been proposed to improve the sensitivity of antiproliferative drugs. In the example of hematopoietic malignancies, apoptotic death of leukemic promyelocytes was achieved by all-trans retinoic acid (ATRA), which induced terminal differentiation [164]. Similarly, Yan et al. demonstrated in breast cancer that ATRA treatment resensitized MDR MCF-7 to epirubicin treatment [165]. In addition, osteopontin is produced by osteoblasts to maintain quiescence in leukemia cells. Neutralization forces dormant leukemia cells to reenter the cell cycle and, crucially, synergistically reduce residual disease in combination with cytarabine chemotherapy [166]. Similarly, treatment with IFNα induces proliferation of dormant hematopoietic SCs, sensitizing them to chemotherapy with 5-fluorouracil [167]. Lastly, an important strategy is to develop drugs that directly kill the dormant cells. For example, in a pancreatic ductal adenocarcinoma model, IGF-1R tyrosine kinase inhibitor, linsitinib, eliminated residual dormant cancer cells with ablation of K-RAS or c-MYC oncogenes [168]. In addition, hydrochloroquine, an autophagy inhibitor, impaired the survival of dormant breast cancer cells, demonstrating a modest effect on metastatic growth [169]. Interestingly, a time sequential treatment of these drugs rather than cotreatment exhibited superior benefit [170,171,172]. For example, Src inhibitors targeting dormant cancer cells improved the efficacy of docetaxel in breast cancer only when administered as a posttaxane treatment but not when the two drugs were administered simultaneously [173]. In summary, targeting dormant CSCs may be a potentially promising strategy; however, its effectiveness remains largely undetermined. Given that no available diagnostic tools for dormant cell detection currently exist, it remains impossible to evaluate the efficacy of this dormant cell–killing approach in patients. 

### 8.4. Targeting CSC Metabolism

**Figure 3:**
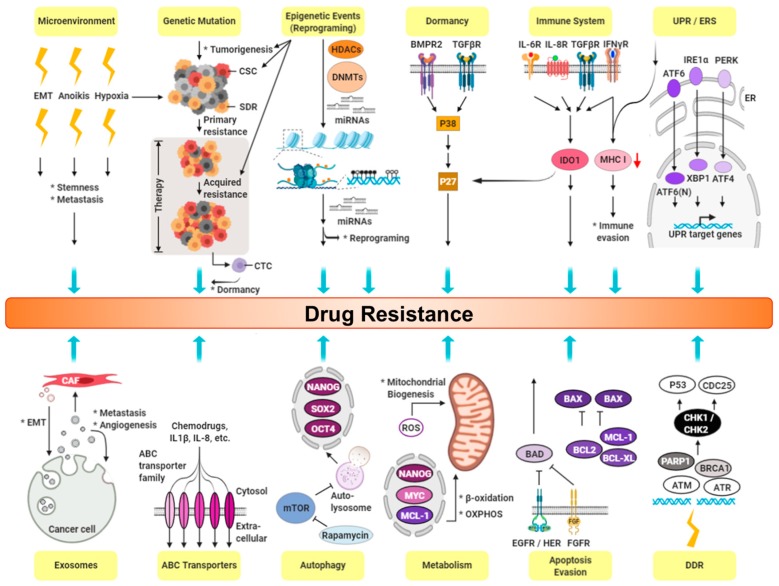
Diagram of molecular pathways involving in CSC drug resistance. System view of drug resistance, which triggered by multiple pathways through subsequent effectors. Some effectors can cross-talk to other molecules leading to stimulate different biological functions, which cooperatively play roles in drug resistance in CSCs. EMT: epithelial-mesenchymal transition; SDR: spontaneous drug resistance; CTC: circulating tumor cell; UPR: unfolded protein response; ERS: endoplasmic reticulum stress; DDR: DNA damage response; ROS: reactive oxygen species; CAF: cancer-associated fibroblast.

The Warburg effect of aerobic glycolysis describes rapidly proliferating cancer cells that overtake angiogenesis, resulting in areas of low oxygen that accelerate glycolytic production of ATP [174]. However, increasing evidence suggests that cancer cells engage in glycolysis even in the presence of oxygen [175,176]. In contrast to normal SCs, which rely heavily on oxidative phosphorylation (OXPHOS) as their primary source of energy, CSCs exhibit unique metabolic flexibility, which primarily entails glycolysis, and can switch between the two in the absence or presence of oxygen to maintain homeostasis; this can promote tumor growth and eventually metastasis [175,177,178,179,180,181]. This observation is supported by studies that have demonstrated that BCSCs consume more glucose, produce less lactate, and have higher ATP content compared with their differentiated progeny [182], providing hints of a possible CSC metabolism-targeted strategy. To date, the inhibition of glycolysis can be achieved by targeting various glycolytic enzymes, transporters, and other complex regulators, such as GLUT1–4, hexokinase, PKM2, and lactate dehydrogenase A [183,184,185]. For instance, the proglyolytic phenotype of BCSCs is associated with decreased expression and activity of pyruvate dehydrogenase (Pdh), a key regulator of OXPHOS; meanwhile, metabolic reprogramming through forced activation of Pdh preferentially eliminates BCSCs both in vitro and in vivo [186]. Moreover, treatment with 2-deoxyglucose, a well-known inhibitor of glycolysis, inhibits BCSC proliferation when used alone and demonstrates a synergic effect when used in combination with doxorubicin [187]. SC markers, such as NANOG, have been implicated in various cancers and have been found to repress mitochondrial OXPHOS genes and ROS generation as well as activate fatty acid oxidation to support CSC self-renewal and drug resistance (Figure 3); however, the restoration of OXPHOS activity and inhibition of fatty acid oxidation renders CSCs susceptible to a standard care chemotherapy drug for hepatocellular carcinoma (HCC), sorafenib [188]. The BCSC marker CD44 interacts with PKM2, enhancing the glycolytic profile of cancer cells deficient in p53 or exposed to hypoxia. Subsequent ablation of CD44 led to the inhibition of glycolysis, an increase in ROS, and the enhancement of chemotherapeutic drug effect in these cancer cells [189,190]. In addition to supporting metabolic plasticity, simultaneous enhancement of glycolysis and OXPHOS pathways was observed in highly metastatic breast cancer cell lines relative to nonmetastatic cell lines [181,191]. Thus, inhibition of glycolytic and/or mitochondrial energy pathways has proven to be effective against tumor growth in a number of preclinical cancer models [187]. Taken together, targeting the CSC metabolism offers considerable potential for developing improved treatments to eradicate them.

### 8.5. Targeting CSC Autophagy

Autophagy homeostasis has been strongly associated with CSC physiology, such as tumorigenesis, differentiation, plasticity, migration/invasion, and pharmacological, viral and immune-resistance [192]. Currently, evidence from breast [193,194], pancreatic, liver [195], osteosarcoma [196], ovarian [197], and glioblastoma [198] CSCs has linked it to autophagy and demonstrated that its suppression negatively affects cell self-renewal capacity. Two key autophagy proteins, BECLIN1 and ATG4, are upregulated in mammospheres when compared to adherent cells, and they are needed for their maintenance and expansion [199,200]. Autophagy has demonstrated the ability to regulate ALDH^+^ BCSCs tumorigenicity through EGFR/STAT3 signaling in a mouse model [201]. Furthermore, recent findings suggest crosstalk between autophagy and EMT [202], chromosome stability [203], cytokines mediation [204], and SC microenvironments [205] in a variety of CSC regulations. Several experimental approaches have revealed that combining cytotoxic drugs and autophagy inhibitors increases CSC sensitivity [206]. For example, chloroquine (a late-stage autophagy inhibitor) enhances drug cytotoxicity in glioblastoma CSCs and reduces their survival when used in combination with EGFR inhibitors or temozolomide [207,208]. Similarly, inhibition of autophagy by chloroquine impairs cell migration and invasion, increases expression of the epithelial marker CD24, and decreases vimentin in BCSCs [208]. Other reagents associated with autophagy-mediated stemness also act in a similar manner. For example, resveratrol-induced autophagy acts on BCSC survival by inhibiting the Wnt pathway [209]. In summary, uncovering the contribution of autophagy to CSC drug resistance remains critical for the development of novel antineoplastic therapies.

### 8.6. Reducing CSC Resistance

Metformin, the most well-known medication for type II diabetes, is a candidate for reducing CSC resistance [210,211]. It has been shown to suppress the BCSC subpopulation, partly through ABC transporter inhibition [211,212]. In addition, Shi *et al*. investigated the use of metformin target KLF5 for degrading and preventing the activation of KLF5 downstream target genes *NANOG* and *FGF-BP1*, which consequently reduced the subpopulation of BCSCs in TNBC [213]. Currently, metformin is used in clinical trials as an adjuvant therapy [214]. Doxycycline, an FDA-approved antibiotic, has been found to reduce bone metastasis and tumor burden in breast and pancreatic cancers [215,216]. Doxycycline was able to reduce CSC resistance to Paclitaxel through the inhibition of mitochondrial biogenesis [217]. Currently, clinical studies are being conducted in advanced breast cancer [NCT01847976].

### 8.7. Targeting CSC-related Niche

The CSC niche regulates intrinsic resistance (Figure 3). Therefore, apart from directly targeting CSCs, instantaneously attacking the CSC microenvironment could be another compelling strategy. Studies have demonstrated that non-CSCs can be reprogrammed to CSCs using EMT [125]. By targeting CAFs and TAMs that secrete EMT-inducing factors, blockage of EMT could be accomplished [91,218]. CAFs play a critical role in the tumor niche, where CSCs maintain high tumorigenicity and cancer stemness with significantly high expression of OCT4 and NANOG. Furthermore, differentiated tumor cells can dedifferentiate into CSCs by being cocultured with CAFs, which suggests signaling crosstalk between CSCs and CAFs; indeed, a paracrine network of IGF/IGF-1R was found to contribute to cancer stemness in the niche environment [219]. IGF-1 is also an obvious serum marker for late-stage endometriosis [220]. Although endometriosis is a nonmalignant disorder, it presents high levels of OCT4 in endometriotic cells [221]. Reportedly, OCT4 is regulated by IGF-1 [110,222,223,224], which suggests that IGF-1 is a critical niche factor for disease progression to an advanced stage of endometriosis. 

Evidence has shown chronic inflammation serves as a protumorigenic factor. High serum levels of IL6, an inflammatory cytokine, can predict the development of hepatitis B virus (HBV)-infected hepatocellular carcinoma (HBV-HCC) [225]. The niche of IL6 stimulates the expression of IGF-1R and autocrinal IGF1 dependently in HBV-HCCs and strongly correlates with OCT4/NANOG expression, early recurrence, and cancer stemness features. Blockage of IL6 and IGF-1R activation disrupts expression of cancer stemness properties in vitro and in vivo, indicating that the inflammatory niche of IL6 promotes early recurrence through IGF-1R activation in patients with HBV-HCC [223]. Furthermore, long-term use of anti-inflammatory anti-cyclooxygenase-2 (COX-2) contributes to 40–50% risk reduction in colon cancer [226]. Some studies have also demonstrated that Celecoxib (COX-2 inhibitor) treatment reduces colorectal CSC subpopulations [227,228]. In addition, treating TNBC cells with IFN*β* suppresses the CSC properties, resulting in decreased tumor sphere formation, EMT expression, and migration, thereby promoting an epithelial phenotype with reexpression of CD24 [229]. Studies have demonstrated the potential of targeting the ECM and its associated proteins. Enzymatic destruction of hyaluronic acid results in stroma reduction and vasculature reexpansion, allowing increased distribution of standard chemotherapy with benefits in an animal pancreatic cancer model [230], and thereby signifying that cancer cells or CSCs are not the only anticancer targets.

Nicotine is a component of tobacco and e-cigarettes, and its receptors (nicotinic acetylcholine receptors, nAChRs) are carcinogens contributing to various cancers. A previous study demonstrated that nicotine not only results in the upregulation of the stemness-related genes OCT4, SOX9, HES1, ALDH1, and STAT3 in pancreatic tumors but also promotes EMT behavior and metastasis [231]. In addition, nicotine also promotes expression of SOX2 and OCT4 through the *α*7-nAChR/YAP1/E2F1 axis in non-small-cell lung cancer (NSCLCs). Tumor cells acquire stemness functions to initiate self-renewal proliferation and migration in NSCLCs [232]. These results strongly support an important role for OCT4 in niche-mediated tumorigenesis and malignancy. Therefore, these molecules and signaling pathways could provide potential strategies for inhibiting nicotine-induced stemness properties and the proliferation of CSCs.

## 9. Promising Targeting Strategies on IGF-1R and nAChRs in TNBC

### 9.1. IGF/IGF-1R Signaling

The IGF signaling system controls energy metabolism, stem cell self-renewal, cell growth, and body size in normal physiology [110,233,234]. Our recent studies on embryonic germline stem cells have demonstrated that IGF1/IGF-1R coordinates hypoxia signals to induce OCT4 expression, maintain normal SC activity, and execute symmetric self-renewal proliferation through HIF-2*α* [110,224]. Moreover, evidence in tumor biology suggests that the IGF signaling pathway is important for tumorigenesis and tumor cell survival and provides chemoresistance ability in several types of cancers, including cancers of the liver, lung, endometrium, prostate, colon, rectum, pancreas, and ovary [222,223,235,236,237,238,239,240,241,242,243]. Patients with breast cancer tend to exhibit high serum levels of IGF-1 [244], and activation of the IGF gene signature has been observed in triple-negative or basal-like breast cancer cell lines [245]. Chang et al. reported that IGF-1 signaling plays an important role in breast cancer progression by controlling both the maintenance of BCSCs and their EMT behavior [246]. Activation of IGF-1R–AKT signaling also elevates CSC properties in platinum-taxol resistant ovarian cancer cell lines [247]. Furthermore, our previous studies have demonstrated that highly expressed IGF-1R was observed in HBV-HCC tissues and was strongly associated with the early recurrence of tumors; furthermore, activation of IGF-1R signaling reduces the susceptibility of tumor cells to chemotherapies; both these outcomes result from induction of critical pluripotent markers, OCT4 and NANOG, by IGF-1R activation in HBV-HCC [222,223]. Therefore, IGF signaling remains a promising therapeutic target for CSCs and chemoresistant diseases.

### 9.2. Nicotine/Nicotinic Acetylcholine Receptors Signaling

Numerous studies have identified the distinguished nAChRs subtypes as mediating tobacco-related cancer development and progression. The nAChRs transduce various signaling cascades upon activation, which govern several pathological conditions, namely tumorigenesis [248,249,250,251,252,253,254,255], metastasis [250,256,257,258,259,260], drug resistance [249,261,262,263], and cancer stemness [263,264,265,266], during carcinogenesis. The homo-pentamer of the *α*7-nAChR subtype is associated with lung [250,255], bladder [253,261], colon [267,268,269], gastric [257,259], and pancreatic [258,262,264] cancer pathologies. In breast cancer cells, the α9-nAChR subtype is the most validated [252]. Studies have identified that α9-nAChR has a role in tumor carcinogenesis in vivo and nicotine-induced transformation of normal human breast epithelial cells in vitro [248,252,270,271]. Moreover, a signaling cascade involving galectin-3, *α*9-nAChR, and STAT3 regulated the enrichment of side population cells with CSC-like properties [263]. Therefore, exposure to nicotine has been correlated with CSC regulation [263,264,265,266]. Most recently, our work on membrane protein communities and cancer membrane protein-regulated networks discovered 13 new interaction proteins (e.g., ERBB2) with *α*9-nAChR across human cancers [272]. In addition, further experiment has revealed that the FDA-approved drug bupropion, which targets *α*9-nAChR, acts as an antimetastasis agent in nicotine-induced breast cancer. In summary, these results indicate the crucial role of *α*9-nAChR in the identification of biomarkers and therapeutic targets and agents.

## 10. Precision Treatment of TNBC

In addition to molecular approach, there are several functional profiling approaches to establish precision treatment strategies against TNBC. Heiser et al. have studied that the variant responses to 77 therapeutic compounds are occurred across numerous breast cancer cell lines including TNBC subtype, and approximately one third showing these specific responses depend on subtype, pathway, and/or genomic aberration [273]. Since last decade, several molecular profiles with measurements of the omics and biological therapeutic responses have enabled the identification of distinctive features that can predict therapeutic response [274,275]. Notably, therapeutic responses are regulated at multiple levels in the genome of different individuals. Based on the patient’s transcriptional subtype, the response rates of precision treatments could be improved [276]. The so-called functional subtyping of breast cancer allows the screening of clinical significance from the wealth of molecular profiling data, speeding the emergence of personalized therapeutic regimens. Extending the work to TNBC, Gautam et al. have studied the responses of 301 approved and investigational oncology compound in 16 TNBC cell lines applying a functional profiling approach. This work revealed that certain levels of protein markers associated with cytotoxic response might serve as markers of response in clinical settings [277]. Furthermore, by utilizing a multiplexed readout for both cell viability and cytotoxicity, a defined spectrum of cellular responses both to single agents and novel combinations in TNBC has been identified. Moreover, the advancement of machine learning for efficient identification of breast cancer-selective therapeutic targets could be the next step for identifying precision medicine approaches in the future [278]. Taken together, systems biology and omics strategies for matching patient cohorts could efficiently respond to new therapies in TNBC, as well as in other types of cancers.

## 11. Conclusions

In this review, we pointed out how multiple mechanisms underlying CSC maintenance give rise to not only tumor survival but also plasticity to drug resistance, particularly under unfavorable stress (Figure 1, Figure 2 and Figure 3). Current clinical practice and ongoing trials on TNBC were discussed systemically, including targeted therapy and subtype-based therapeutic strategies. Agents that target various aspects of CSCs, namely specific markers, signaling, dormancy, proliferation, metabolism, autophagy, drug resistance, and niches, which have generated promising preclinical results and are now entering clinical trial, were reviewed. We hope our work provides a better understanding of CSC pathology and encourages scientists to engage in more creative attempts to develop more effective strategies to achieve better treatment outcomes in the future.

The considerable disease heterogeneity, both intertumor and intratumor, remains the major obstacle to identifying actionable targets in TNBC. Due to the lack of well-defined molecular targets, current treatment options for TNBC commonly focus on cytotoxic chemotherapy. Despite different TNBC subtypes presenting distinct treatment responses, subtype-based chemo regimen selection remains debatable. In addition, cytotoxic chemotherapy against actively dividing cells within a tumor is not capable of eliminating dormant CSCs, which results in low pCR and short DFS in patients with TNBC. Although more aggressive chemotherapy has resulted in improved prognosis, it usually leads to serious deterioration in quality of life. This has prompted endeavors to develop novel drugs and identify effective subtype-specific therapeutic strategies, such as anti-IGF-1R and anti-*α*9-nAChR in TNBCs (Figure 2). 

Several studies over a number of years have examined target therapies against CSCs in hopes of eradicating MDR. However, only targeting these CSCs does not succeed in tumor eradication either. While “one size does not fit all TNBCs” is the lesson learned from the past, the same rule applies in so far as the previous failure in CSC-targeted therapy does not determine all cases. Recent studies have demonstrated satisfying results when they have repositioned a CSC-targeted regimen in other breast cancer prescriptions, such as in combination with chemotherapy or time-sequential treatment rather than co-treatment. Moreover, anti-IGF-1R or anti-*α*9-nAChR are reasonable strategies to combine with chemotherapies to attack the CSCs of TNBCs (Figure 2). Given the increasing knowledge of the relevance of the dynamic and changing nature of cancer and CSC populations, we predict that tailored therapeutic strategies alongside TNBC treatment will be the future gold standard. 

## Figures and Tables

**Table 1:** The ongoing clinical trials in triple-negative breast cancer.

Target	Major Drug	Combinational Drug	Phase	NCT Identifiers	Status ^#^
**BRCA1/2**					
PARP	BGB290	± Temozolomide	I/II	NCT03150810	R
	BMN-673	-	II	NCT02401347	R
		± Carboplatin + Paclitaxel	I	NCT02358200	Ac/NR
		± CB-839 (GLS1i)	I/II	NCT03875313	R
		± ZEN003694 (BETi)	II	NCT03901469	R
	Niraparib	± Pembrolizumab	I/II	NCT02657889	Ac/NR
	Olaparib	-	II	NCT00679783	Ac/NR
		± AZD2014 (mTORi) ± AZD5363 (AKTi)	I/II	NCT02208375	Ac/NR
		± AZD6738 ± AZD1775	II	NCT03330847	R
		± BKM120 (PI3Ki) ± BYL719 (PI3Ki)	I	NCT01623349	Ac/NR
		± Carboplatin ± Paclitaxel	I	NCT00516724	Ac/NR
		± Carboplatin ± Paclitaxel	II/III	NCT03150576	R
		± Cediranib (VGEFi)	I/II	NCT01116648	Ac/NR
		± Cediranib ± Durvalumab	I/II	NCT02484404	R
		± Durvalumab (anti PD-1)	I	NCT03544125	R
		± Durvalumab (anti PD-1)	II	NCT03801369	R
		± Durvalumab (anti PD-1)	II	NCT03167619	R
		± Onalespib (HSP90i)	I	NCT02898207	R
	Rucaparib	± Cisplatin	II	NCT01074970	Ac/NR
	Veliparib	± Carboplatin ± Cyclophosphamide ± Doxorubicin ± Paclitaxel	II	NCT01818063	Ac/NR
		± Carboplatin ± Cyclophosphamide ± Doxorubicin ± Paclitaxel	III	NCT02032277	Ac/NR
		± Cisplatin	II	NCT02595905	R
		± Irinotecan Hydrochloride	I	NCT00576654	Ac/NR
		± Lapatinib (EGFR/HER2i)	Pilot study	NCT02158507	Ac/NR
**CHK1 and WEE1**					
CHK1	LY2880070	± Gemcitabine	I /II	NCT02632448	R
CHK1/2	LY2606368	-	II	NCT02203513	R
WEE1	AZD1775	-	I	NCT02482311	Ac/NR
		± Cisplatin	II	NCT03012477	Ac/NR
**Cyclin-dependent kinases**					
CDKs	Abemaciclib	-	II	NCT03130439	R
	PF-06873600	± Endocrine Therapy	II	NCT03519178	R
	Ribociclib	± Bicalutamide (ARi)	I/II	NCT03090165	Ac/NR
	SHR6390	± SHR3680 (ARi)	I/II	NCT03805399	R
	Trilaciclib	± Gemcitabine + Carboplatin	II	NCT02978716	Ac/NR
**Androgen receptor**					
Androgen	Bicalutamide	-	III	NCT03055312	R
	CR1447(ARM)	-	II	NCT02067741	Ac/NR
	Darolutamide (NSAA)	± Capecitabine	II	NCT03383679	R
	Enzalutamide	-	II	NCT02750358	Ac/NR
		-	II	NCT01889238	Ac/NR
		± Paclitaxel	II	NCT02689427	R
	GTx-024 (SARM)	± Pambrolizumab (anti PD-1)	II	NCT02971761	R
**Growth factors and angiogenesis**					
EGFR	Cetuximab	± Ixabepilone	II	NCT01097642	Ac/NR
	Icotinib	-	II	NCT02362230	R
	Panitumumab	± Gemcitabine + Cisplatin	II	NCT02546934	R
		± Carboplatin + Paclitaxel	II	NCT02593175	R
		± Carboplatin + Paclitaxel + Doxorubicin + Cyclophosphamide	II	NCT02876107	R
	SCT200	-	II	NCT03692689	R
HER2	Trastuzumab	± Paclitaxel + Cyclophosphamide	II	NCT01750073	R
VEGF	Bevacizumab	± Paclitaxel + Erlotinib (EGFRi)	II	NCT00733408	Ac/NR
VEGFR2	Apatinib	± Fluzoparib (PARPi)	I	NCT03075462	R
		± Vinorelbine	II	NCT03254654	R
**SRC and WNT signaling**					
SRC	Dasatinib	-	II	NCT02720185	R
WNT	LGK974	± PDR001 (anti-PD-1)	I	NCT01351103	R
		± Gedatolisib (PI3Ki)	I	NCT03243331	R
**PI3K/AKT/mTOR pathway**					
PIK3CA	Taselisib	± Enzalutamide (ARi)	I/II	NCT02457910	R
PIK3CB	IPI-549	± Nivolumab (anti PD-1)	I	NCT02637531	R
	PQR309	± Eribulin	I/II	NCT02723877	R
PI3K	AZD8186	-	I	NCT01884285	R
		± Docetaxel	I	NCT03218826	R
	BKM120	± Capecitabine + Trastuzumab (HER2i)	II	NCT02000882	Ac/NR
	CUDC-907	-	I	NCT02307240	Ac/NR
AKT	ARQ092	± Carboplatin + Paclitaxel	Ib	NCT02476955	R
	AZD5363	± Paclitaxel	II	NCT02423603	Ac/NR
	GSK2141795	± Trametinib (MEKi)	II	NCT01964924	Ac/NR
	Ipatasertib	-	II	NCT02162719	Ac/NR
		± Carboplatin + Paclitaxel	I/II	NCT03853707	R
		± Paclitaxel	II/III	NCT03337724	R
mTOR	AZD2014	± Selumetinib (ERKi)	I/II	NCT02583542	Ac/NR
	Everolimus	± Cisplatin + Paclitaxel	I	NCT02120469	Ac/NR
		± Cisplatin + Paclitaxel	II	NCT02531932	R
		± Doxorubicin + Bevacizumab (VEGFi)	II	NCT02456857	R
	Gedatolisib	± Docetaxel + Cisplatin + Dacomitinib (EGFRi)	I	NCT01920061	R
		± Tak-117 (PI3Ki) + Cisplatin + Paclitaxel	II	NCT03193853	R
**Immune checkpoint**					
PD-1	Nivolumab	± Capecitabine	II	NCT03487666	R
		± Carboplatin	I	NCT03414684	R
	PDR001	-	I/II	NCT02404441	Ac/NR
	Pembrolizumab	-	I	NCT03197389	R
		-	II	NCT02447003	Ac/NR
		-	II	NCT02644369	Ac/NR
		-	III	NCT02555657	Ac/NR
		± Capecitabine ± Paclitaxel	I/II	NCT02734290	R
		± Doxorubicin ± Aromatase inhibitors	II	NCT02648477	R
		± Nab-paclitaxel (or Paclitaxel) + Carboplatin + Doxorubicin + Cyclophosphamide	I	NCT02622074	Ac/NR
		± Nab-paclitaxel (or Paclitaxel) ± Gemcitabine + Carboplatin	III	NCT02819518,	Ac/NR
		± Paclitaxel + Carboplatin ± Doxorubicin ± Epirubicin + Cyclophosphamide	III	NCT03036488	Ac/NR
		± Lenvatinib (TKI)	II	NCT03797326	R
		± PVX-410 Vaccine	I	NCT03362060	R
	SHR1210	± Apatinib (TKI)	II	NCT03394287	Ac/NR
PD-L1	Atezolizumab	± Carboplatin	II	NCT03206203	R
		± Carboplatin + Cyclophosphamide ± Paclitaxel	II	NCT01898117	R
	Durvalumab	± Oleclumab + Paclitaxel + Carboplatin	I/II	NCT03616886	R
		± Paclitaxel	I/II	NCT02628132	R
		-	II	NCT02685059	Ac/NR
	FAZ053	± PDR001	I	NCT02936102	Ac/NR

#, R: recruiting; Ac/NR: active, not recruiting.

**Table 2:** The ongoing clinical trials of potential targeting molecules in carcinoma diseases.

Target	Major Drug	Combinational Drug	Indication	Phase	NCT Identifiers	Status ^#^
**Receptor**						
IGF-1R	AMG479 (Ganitumab)	± Dasatinb	Embryonal and alveolar rhabdomyo-sarcoma	I/II	NCT-03041701	R
		± (Everolimus + Panitumumab)	Advanced solid tumors, NSCLC	I	NCT-01061788	Ac/NR
		± Vincristine + Doxorubicin + Cyclo-phosphamide / Ifosfamide + Etoposide	Metastatic malignant neoplasm in the bone, bone marrow, lung, and etc.	III	NCT-02306161	Ac/NR
	Cixutumumab	± Lapatinib ditosylate + Capecitabine	Breast cancer	II	NCT-00684983	Ac/NR
		± Paclitaxel	Esophageal cancer, Gastro-esophageal junction adeno-carcinoma	II	NCT-01142388	Ac/NR
	DNA Plasmid Based Vaccine (WOKVAC)	-	Breast cancer	I	NCT-02780401	R
	MK-0646 (Dalotuzumab)	± Gemcitabine + Erlotinib	Advanced pancreatic cancer	I/II	NCT-00769483	Ac/NR
	Teprotumumab	-	Thyroid eye disease, Graves’ orbitopathy	III	NCT-03298867	Ac/NR
	Trastuzumab	± Lapatinib ditosylate	Breast cancer	III	NCT-01104571	Ac/NR
nAChR	Bupropion	-	Breast cancer	III	NCT-03996265	R
**Cytokine**						
IL-6 receptor	Tocilizumab	± Ipilimumab + Nivolumab	Advanced melanoma	II	NCT-03999749	R
IL-7 receptor	Tocilizumab	± Gemcitabine + nab-Paclitaxel	Unresectable pancreatic carcinoma	II	NCT-02767557	R
**Dormancy and proliferation**						
	All-trans retinoic acid (ATRA)	± 5-Azacitidine	Prostate cancer	II	NCT-03572387	R
**Metabolism**						
Glycolysis inhibition	2-Deoxyglucose (2DG)	-	Advanced cancer and hormone refractory prostate cancer	I/II	NCT-00633087	T
**Autophagy pathway**						
Autophagy inhibition	Hydro-chloroquine	-	Breast cancer	II	NCT-01292408	U
	Chloroquine	± Carboplatin + Gemcitabine	Advanced solid tumors	I	NCT-02071537	R
		+ Temozolomide + Radiotherapy	Glioblastoma multiforme	I	NCT-02378532	R
**Drug resistance**						
Resistance inhibition	Doxycycline	-	Bone-targeted therapy in patients with metastatic breast cancer	II	NCT-01847976	U
		-	Cutaneous T-cell lymphoma	II	NCT-02341209	R
	Metformin	-	Colon cancer	II	NCT-03359681	NR
		-	Prostate cancer	II	NCT-03137186	R
		-	Prostate cancer	II	NCT-02176161	R
		-	Breast cancer prevention	III	NCT-01905046	R
		+ Doxycycline	Breast and uterine corpus cancer	II	NCT-02874430	R
		+ Erlotinib	TNBC	I	NCT-01650506	C
		+ Paclitaxel + Carboplatin + Docetaxel	Ovarian, primary peritoneal, or fallopian tube carcinoma	II	NCT-02122185	R
		+ Pembrolizumab	Melanoma	I	NCT-03311308	R
		+ Temozolomide	Glioblastoma	II	NCT-03243851	
		+ Temsirolimus	Advanced cancers	I	NCT-01529593	Ac/NR
**Others**						
CD44	RO5429083	-	CD44-expressing, malignant solid tumors	I	NCT-01358903	C
ER	Tamoxifen	+ 9-Cis-retinoic acid	Breast cancer	I	NCT-00001504	C
GD2	3F8/GM-CSF	+ 13-Cis-retinoic acid	Primary refractory neuroblastoma in bone marrow	II	NCT-01183897	Ac/NR
Hedgehog	Vismodegib	-	Basal cell carcinoma	II	NCT-03035188	R
		+ RO4929097	Breast cancer	I	NCT-01071564	T

#, R: recruiting; Ac/NR: active, not recruiting; T: terminated; U: unknown; C: completed; NR: not yet recruiting.
